# Good Manufacturing Practice Validation and Radiation Dosimetry for the Clinical Application of a Novel α7-nAChR Radioligand: [^11^C]KIn83

**DOI:** 10.3390/molecules30061356

**Published:** 2025-03-18

**Authors:** Zhisheng Jia, Martin Bolin, Anton Forsberg Morén, Prodip Datta, Heba Asem, Hans Ågren, Bengt Långström, Agneta Nordberg, Christer Halldin, Sangram Nag

**Affiliations:** 1 Department of Clinical Neuroscience, Centre for Psychiatry Research, Karolinska Institutet and Stockholm County Council, 171 77 Stockholm, Sweden; zhisheng.jia@ki.se (Z.J.); martin.bolin@ki.se (M.B.); anton.forsberg.moren@ki.se (A.F.M.); prodip.datta@ki.se (P.D.); heba.asem.ismail@ki.se (H.A.); sangram.nag@ki.se (S.N.); 2 Division of X-ray Photon Science, Uppsala University, 751 20 Uppsala, Sweden; hans.agren@physics.uu.se; 3 Department of Chemistry, Uppsala University, 751 20 Uppsala, Sweden; bengt.langstrom@kemi.uu.se; 4 Division of Clinical Geriatrics, Center for Alzheimer Research, Department of Neurobiology, Care Sciences and Society, Karolinska Institutet, 141 52 Stockholm, Sweden; agneta.k.nordberg@ki.se; 5 Theme Inflammation and Aging, Karolinska University Hospital, 141 52 Stockholm, Sweden; 6 HUN-REN TKI, Department of Biophysics and Radiation Biology, Semmelweis University, 1094 Budapest, Hungary

**Keywords:** nicotinic acetylcholine receptor, Alzheimer’s disease, non-human primate, dosimetry, PET

## Abstract

Nicotinic acetylcholine receptor (α7-nAChR) plays a crucial role in cognitive functions like memory and attention. Positron emission tomography (PET) imaging of α7-nAChR is gaining attraction for understanding and monitoring central nervous system disorders, such as Alzheimer’s disease, Parkinson’s disease, and schizophrenia. We developed [^11^C]KIn83, a novel α7-nAChR radioligand, and evaluated its biological properties. This study focused on two objectives: (1) to validate its Good Manufacturing Practice (GMP)-compliant production, and (2) to assess the dosimetry of [^11^C]KIn83 using non-human primate (NHP) whole-body PET data. Radiolabeling and drug product delivery of [^11^C]KIn83 were conducted using an automated synthesis module within a controlled GMP environment. The quality control tests performed adhered to the European Pharmacopoeia guidelines. The production of [^11^C]KIn83 was validated according to GMP standards, encompassing automated synthesis and quality control measures. For the dosimetry assessment, two female cynomolgus monkeys underwent whole-body PET scans. The radioactivity values injected for [^11^C]KIn83 were 150 MBq and 155 MBq, respectively, with an estimated radiation dose of 0.0047 mSv/MBq. Our findings pave the way for future clinical studies that investigate the potential of [^11^C]KIn83 to measure α7-nAChR, aiding our understanding and possibly supporting diagnoses of different cognitive disorders.

## 1. Introduction

Molecular imaging using PET enables the visualization and quantification of various biological processes in living organisms with high sensitivity and specificity. In the realm of neuroscience, the development of PET tracers targeting α7-nicotinic acetylcholine receptors has emerged as a promising avenue for elucidating the role of these receptors in normal brain function and disease states [1]. α7-nAChRs are a subtype of nicotinic acetylcholine receptors that are widely distributed throughout the central nervous system and play a critical role in cognitive processes, learning, memory, and synaptic plasticity [2]. The dysregulation of α7-nAChRs has been implicated in various neuropsychiatric disorders, including Alzheimer’s disease, schizophrenia, and depression, underscoring the importance of studying these receptors in both healthy and disease subjects [3,4]. The development of PET tracers targeting α7-nAChRs offers an opportunity to non-invasively investigate the distribution, density, and function of these receptors in the living brain. By selectively binding to α7-nAChRs, these radiotracers provide valuable insight into receptor occupancy, regional expression patterns, and pharmacological interactions in real time [5]. Furthermore, the PET imaging of α7-nAChRs holds great promise for advancing our understanding of neurological and neuropsychiatric disorders at the molecular level. Through the precise quantification of α7-nAChR availability and kinetics, researchers can explore the pathophysiological mechanisms underlying disease progression in order to evaluate treatment responses and to potentially identify novel therapeutic targets for intervention [6]. In this work, we set the stage for exploring the development and applications of PET tracers targeting α7-nAChRs. By delving into the methodologies of preclinical and clinical studies and future directions in this burgeoning field, we aim to shed light on the transformative potential of α7-nAChR-targeted PET imaging in unraveling the complexities of brain function and dysfunction. Numerous PET ligands, such as [^18^F]ASEM [7], [^11^C]NS14492 [8], [^11^C]CHIBA-1001 [9], [^18^F]DBT-10 [5], and [^11^C]A-582941 [10], have been developed for selectively targeting α7-nicotinic acetylcholine receptors (α7-nAChRs).

Developing PET radioligands is complex [11], demanding a meticulous approach to ensure safety and efficacy. This requirement necessitates a deep understanding and strict implementation of three components: process validation, CMC (Chemistry, Manufacturing, and Controls), and dosimetry experiments. These elements are not simply separate steps; they form an interwoven foundation for developing reliable and clinically relevant PET radioligands, facilitating their successful translation from the laboratory to clinical applications and research.

Process validation and CMC are integral components of radiochemistry in the production of PET radioligands. The validation of processes and adherence to CMC guidelines are essential for ensuring the quality, safety, and efficacy of PET radioligands intended for molecular imaging studies [12]. Process validation in radiochemistry entails that each stage of radioligand synthesis, purification, and formulation can be consistently reproduced to meet established quality standards. By implementing validation procedures, radiochemists can ensure the reliability and reproducibility of PET radioligands, thereby enhancing the precision and validity of neuroimaging investigations.

Dosimetry experiments play a fundamental role in the development of PET radioligands, providing valuable insights into the radiation levels associated with them. As PET imaging involves the administration of radioactive compounds for diagnostic purposes, understanding the dosimetry profile of a radioligand is essential for ensuring patient safety, optimizing imaging protocols, and complying with regulatory guidelines. In the context of PET radioligand development, dosimetry experiments aim to quantify the radiation doses absorbed by various organs and tissues following the administration of a radiotracer [13,14]. By measuring the biodistribution, pharmacokinetics, and clearance of the radioligand in preclinical models, we can estimate the radiation doses received by different body regions over time. These dosimetry assessments are critical for evaluating the potential radiation risks associated with using a specific PET radioligand in humans, guiding dose optimization strategies, and determining appropriate imaging protocols. Moreover, dosimetry data informs regulatory authorities about the safety of the radioligand, facilitating its approval for clinical use and allowing radiation exposure in patients during PET imaging procedures to be monitored.

Building on previous work, which detailed the development and characterization of a novel α7-nAChR radioligand [^11^C]Kln83 [15], we now present the findings of a comprehensive study aimed at advancing its potential for clinical translation. Our objectives were as follows:

(i) GMP-compliant Production Verification: To pave the way for potential clinical use, we rigorously verified the Good Manufacturing Practice (GMP) compliance of the [^11^C]Kln83 production process. This validation encompassed a comprehensive review of all aspects of the manufacturing process, from raw materials and synthesis procedures to quality control measures and final product specifications. Meeting GMP standards ensures consistent production of a high-quality, safe, and efficacious radiopharmaceutical, which is vital for its successful transition into clinical settings.
(ii) Dosimetry Assessment: To ensure the safe and effective application of [^11^C]Kln83 in human studies, we conducted dosimetry assessments using data obtained from whole-body PET scans in non-human primates (NHPs). This crucial step involved meticulously evaluating the distribution and clearance of the radioligand within the NHPs’ bodies, allowing us to calculate the radiation dose received by various organs and tissues. The data obtained from these assessments will be essential for determining the optimal dosage for human studies, minimizing radiation exposure while maximizing diagnostic efficacy.

## 2. Results and Discussion

### 2.1. Radiochemistry, Validation, and CMC

**Scheme 1:**
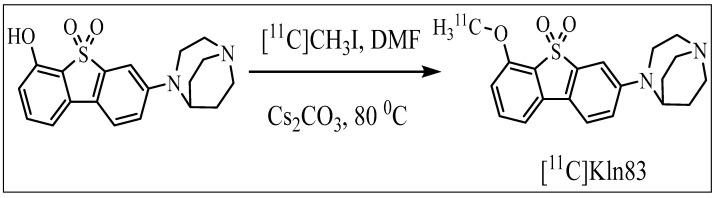
Synthesis of [^11^C]KIn83.

The synthesis of [^11^C]KIn83 was set up in a fully automated GMP-complaint synthesizer, “TRACERlab FX2 C Synthesizer”, supplied by GE. The radiolabeling was achieved via the classical one step *O*-methylation of the corresponding desmethyl precursor (Scheme 1).

Three batches of [^11^C]KIn83 solution for injection were manufactured during the process of validation; quality control focused on both the pre-release and post-release state of the batches. Moreover, a bioburden test batch was purposefully produced without sterile filtration to assess bioburden contamination in a worst-case scenario. Due to the short half-life of carbon-11, the filter screening, determination of pH, and assessment of the chemical and radiochemical purity were performed with the limitation of taking less than 20 min.

The average yield of the process was 622 MBq (650 MBq, 697 MBq, and 520 MBq, respectively), and the radiochemical purity of each batch exceeded 95%. Throughout these tests, the resulting product consistently appeared colorless and transparent, with no visible particles. The pH of the solution remained stable between 6.0 and 7.0, which is compatible with intravenous injections. UV-absorbing impurities were below the limit of detection in all validation batches (n = 3). With the help of the proper functioning of the filter integrity test, the final sterilization process of the solution was achieved. During the validation, the precursor was not detected in any of the batches, remaining below the limit of detection. Bacterial endotoxin testing showed that all batches contained levels below 3.5 IU/mL, indicating minimal contamination. Furthermore, all four batches, including the bioburden batch, were confirmed to be sterile. The amount of residual acetonitrile, acetone, and DMF in the produced validation batches was within the specifications. The ethanol content was below 10% in all produced batches, including the bioburden batch. Moreover, the estimated quantity of [^11^C]KIn83 per dose, calculated at 30 min after synthesis using a dose of 300 MBq, was consistently below 5 μg across all batches. For a detailed overview of the validation results, please see Table S1.

The chemistry properties of KIn83, as well as the organic synthesis, manufacturing process, process control, and their results are described under ‘chemistry manufacturing and controls’ in detail.

### 2.2. Whole-Body PET

**Figure 1:**
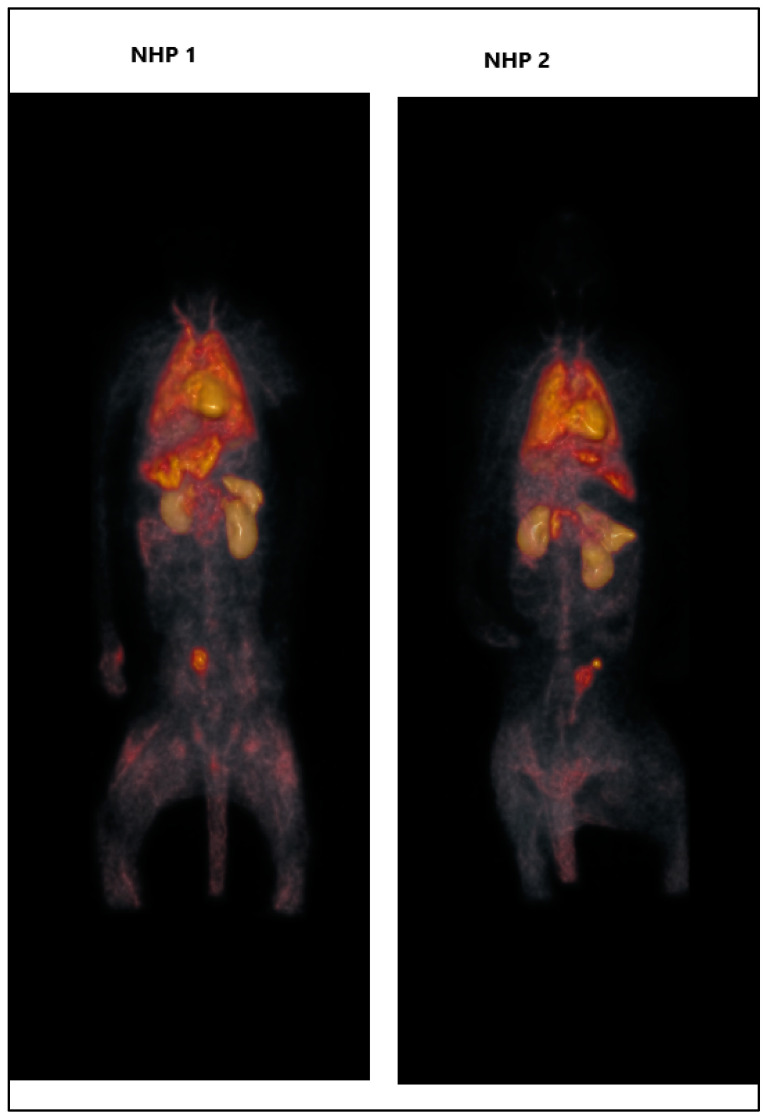
Whole-body PET images of [^11^C]Kln83 in NHP1 and NHP2.

**Figure 2:**
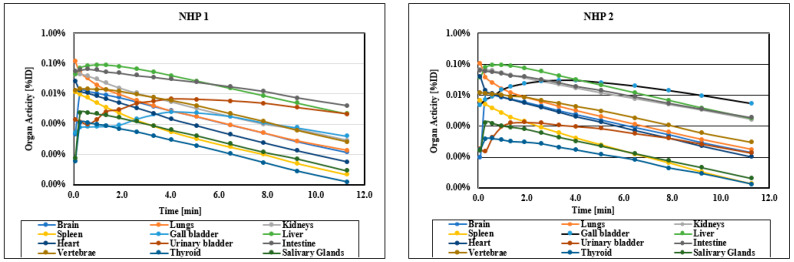
Whole-body time–activity curves for [^11^C]KIn83 in different regions.

The injected radioactivity of [^11^C]KIn83 was 150 MBq for NHP1 and 155 MBq for NHP2. Figure 1 shows maximum intensity projection (MIP) PET image for NHP1, while Figure 2 depicts the time activity data of both NHP1 and NHP2. The level of uptake of [^11^C]KIn83 was in general comparable between the two non-human primates (NHPs). The volumes of the selected regions of interest, which include the brain, heart, liver, lungs, kidneys, gallbladder, bone (lumbar vertebrae), urinary bladder, stomach, small intestine, spleen, and pancreas, are presented in Table 1. The radioligand shows notable uptake, primarily localized in the excretory system (liver, small intestine, gallbladder, urinary bladder), before being distributed in the brain and kidneys.

Table 2 displays the number of disintegrations in the source organs. Among these, the liver received the highest absorbed dose, measured at 0.014 mGy per megabecquerel (MBq) (see Table 3, Figure S1:a-l). The estimated effective dose (ED) was calculated as 0.0047 mSv per MBq (as shown in Table 4), which is relatively low for carbon-11 PET radioligands [16,17]. This low ED is likely due to the minimal uptake in the bladder, which subsequently reduces the absorbed dose to nearby radiosensitive organs. This indicates that an injection of 400 MBq of [^11^C]KIn83 can be administered, at multiple occasions, to humans if the maximum allowable dose for total radiation exposure is 10 mSv. The radiation exposure caused by [^11^C]KIn83 is comparable to other carbon-11 ligands, as has been extensively compared previously [18].

## 3. Materials and Methods

GE HealthCare provided the equipments, including a fully automated synthesizer (TRACERlab™ FX2 C) and cyclotron. The purification of the radioligand was conducted using a semi-preparative reverse phase (RP) ACE column (C18, 10 × 250 mm, 5 µm particle size) and a Merck Hitachi UV detector (λ = 254 nm) (VWR International, Stockholm, Sweden) connected to a GM tube (USA, Carroll-Ramsey, Berkeley, CA), which was utilized for the detection of radioactivity. Syngene International Ltd., India, supplied the corresponding desmethyl precursor (3-(1,4-diazabicyclo [3.2.2]nonan-4-yl)-6-hydroxyl-dibenzo[b,d] thiophene 5,5-dioxide) and the reference standard of KIn-83 (3-(1,4-diazabicyclo [3.2.2]nonan-4-yl)-6–11C-methoxydibenzo[b, d] thiophene 5,5-dioxide). All other chemicals were obtained from the suppliers and utilized as such without further purification. Solid-phase extraction (SPE) cartridges (Sep-Pak tC18 1 cc cartridges) were from Waters (USA, Milford, MA). The activation of the C18 Plus cartridge was carried out with 10 mL of ethanol (EtOH), followed by washing with 10 mL of sterile water. Sterile filters (Millex-GV, 0.22 µm) and vent filters (Millex-FG, 0.20 µm, hydrophobic PTFE) were purchased from Merck-Millipore (Burlington, MA, USA).

### 3.1. Synthesis of [^11^C]KIn83

[^11^C]KIn83 was synthesized using a one-pot ^11^C-methylation procedure with [^11^C]methyl iodide ([^11^C]CH_3_I), following a previously published method [15]. In short, carbon-11-labeled methane ([^11^C]CH_4_) was produced in the target through the nuclear reaction ^14^N(p,α)^11^C, using nitrogen containing hydrogen (10%) and 16.5 MeV protons from the GEMS PET Trace cyclotron (GE, Uppsala, Sweden). The resultant [^11^C]CH_4_ was condensed into cooled Porapak and later evacuated from the adsorbent. The [^11^C]CH_4_ was first mixed with vapor of iodine in a closed circulating system, and a radical reaction proceeded at 720 °C in a quartz tube. The resultant [^11^C]CH_3_I was absorbed on Porapak Q at ambient room temperature and desorbed by step warming to 180 °C in the presence of helium gas.

The [^11^C]KIn83 synthesis was performed by trapping [^11^C]CH_3_I at room temperature inside a vial containing desmethyl precursor (0.50 mg, 1.2 µmol) and cesium carbonate (5,0 mg) in dimethylformamide (DMF) (500 µL) (Scheme 1). After the completion of the trapping, the reaction mixture was heated for 3 min at 80 °C. The crude mixture was diluted with HPLC mobile phase (300 µL) and injected into an integrated high-performance liquid chromatography (HPLC) system equipped with an ACE semi-preparative reverse phase (RP) column (C18 10 × 250 mm 5µm) and a Merck Hitachi UV detector (λ = 254 nm) in series with a GM tube for radioactivity detection. A mobile phase of acetonitrile and 0.1 M ammonium formate (35:65, *v:v*) with a flow rate of 6 mL/min was used to elute the appropriate radioactive fraction corresponding to the desired product, [^11^C]KIn83. The radioactive fraction collected via HPLC was diluted with sterile water (50 mL). The resulting mixture was loaded through a pre-conditioned (10 mL ethanol followed by 10 mL sterile water) SepPak tC18 1cc cartridge. The cartridge was washed with sterile water (10 mL) and then the isolated product in the SPE cartridge was eluted out with ethanol–propylene glycol solution, which contains 0.1% polysorbate. This product was then reformulated with sterile saline (0.9%). The resulting solution contained less than 10% absolute ethanol and was sterilized by means of membrane filtration.

### 3.2. Process Validation of [^11^C]KIn83

In order to ensure and uphold superior quality and safety standards, the [^11^C]KIn83 radiopharmaceutical underwent a comprehensive validation process that met the conditions of current Good Manufacturing Practices (cGMPs) [19]. All recommendations of the European Pharmacopoeia were carried out, which thereby aided in ensuring that the quality control tests, and the product specifications were in order.

**Figure 3:**
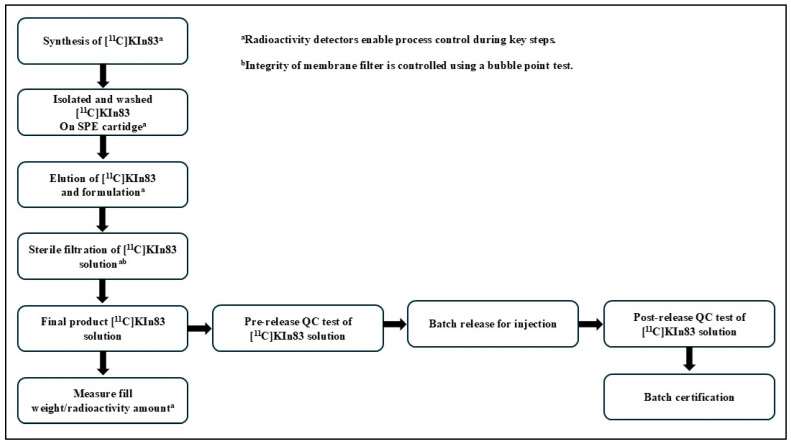
Flow diagram of the manufacturing process of [^11^C]KIn83.

The manufacturing process complied with the schematic flowchart in Figure 3, indicating all the processes carried out in manufacturing. After the removal of the HPLC mobile phase, [^11^C]KIn83 was formulated in a saline solution containing no more than 10% ethanol, propylene glycol (20%), and polysorbate 80 (<0.03%). To retain the sterility of the solution, the solution was directly passed through a 0.22 μm membrane filter into a sterile vial. A bubble point test was performed to verify whether the filtration was successful, and this step was taken as a critical part of the validation of the filter after the filtration process. The final product underwent a thorough assessment with interest in the weight and pH, among other relevant criteria, in order to confirm its compliance with established quality standards. Furthermore, upon successful completion of the pre-administration quality control analysis and product release, the batch of [^11^C]KIn83 solution for injection was ready to be employed in a clinical setting, having satisfied all the important safety and efficacy aspects for patient use.

### 3.3. Quality Control (QC)

The in-house quality check procedures that were applied in the analysis of the [^11^C]KIn83 product were thoroughly validated against the set industrial standards [19,20] to ensure reliability and accuracy (Table 1). The other quality control tests were performed by subcontracted reputable external contractors, as is the custom in the industry. Due to the short half-life of carbon-11 (T1/2~20.3 min), not all quality control tests could be conducted on every single batch produced. Consequently, we established a defined set of pre-release and post-release quality control tests to ensure the quality and reliability of the produced batches (Table 4). This practice makes it possible for all testing procedures to be of the high quality that is deemed necessary to give correct results.

#### 3.3.1. Pre-Release QC Tests

Conducting comprehensive pre-release quality control (QC) for PET radioligands is critical to ensure that the products meet specified standards for safety, efficacy, and regulatory compliance. Below is an outline of typical pre-release QC parameters and processes:

Visual Inspection: Evaluate the radioligand [^11^C]KIn83 solution for clarity, color, and particulate matter.

pH determination: The determination of pH for the product solution was conducted using pH indicator strips (pH 2.0–9.0) from Merck, Germany. This process ensures pH is within an acceptable range for the intended use.

Filter Integrity Test: Assessing the integrity of the sterile filters used during the production process ensures the final product is free from contaminants. Filter integrity was evaluated by bubble-point measurements using the Model 010105280602-A device from DM Automation (Nykvarn, Sweden).

Identity Confirmation by chromatographic Analysis: Perform HPLC to verify the identity of the radioligand, and with the co-injection of the corresponding authentic reference confirm the presence of the correct compound (Figure S1). We used an analytical HPLC (Stockholm, Sweden) system coupled with an ACE 5 C18-HL (100 × 4.6 mm^2^) column to measure radiochemical impurity, product identity, radiochemical stability, and chemical purity for product verification. A Merck-Hitachi L-7100 pump, an L-7400 UV detector, and a GM tube for radioactivity detection (VWR International) were mounted. The gradient mobile phase for HPLC comprised 20% acetonitrile in ammonium formate solution (0.1 M) to 60% acetonitrile in ammonium formate for 4 min, continued for 1 min at same composition at a flow rate of 1.2 mL/min. Constant monitoring of the HPLC liquid flow was accomplished with a combination of a UV absorbance detector (λ = 263 nm) and a radioactive detector (BETA-flow, Beckman, Fullerton, CA).

Radiochemical Purity: Radiochemical purity was analyzed using HPLC and radioactivity detection.

Radionuclidic identity: The radionuclidic identity of [^11^C]KIn83 is established by assessing the half-life of carbon-11 using a dose calibrator.

Chemical Purity: Confirm that non-radioactive components in the final product are within acceptable limits, as determined by HPLC analysis method.

Radioactive Stability: Assess the stability of the radioligand at designated time points (e.g., 30 and 60 min) post-synthesis to ensure radiochemical purity and integrity over time. The analysis was performed using HPLC.

Bacterial Endotoxin Testing: Conduct testing using a validated system (e.g., Endosafe^®^ Nexgen-PTS) to quantify the presence of bacterial endotoxins and ensure product safety. The dilution factor 70 is used for the optimal analysis of the bacterial endotoxin content of the sample.

#### 3.3.2. Post-Release QC Tests

Residual Solvent Testing: HPLC was used to measure the residual DMF (Figure S2), whereas gas chromatography (GC) was applied to assess residual solvents, ethanol, acetonitrile, and acetone (Figure S3). The GC analysis was performed by employing an Agilent Technologies 6850 series GC system with a flame ionization detector, FID, and a Res-Solv column (30 m x 0.53 mm, i.d 1.0 µm). An auto-injector and head-space sampler (Model: G4513A, Agilent Technologies) were used for sample introduction. The HPLC (Ultimate/Thermofisher) system was equipped with a Waters Atlantis T3 5 µm (150 × 4.6 mm^2^) column to identify and determine the content of DMF as residual solvent (Figure S4). A Merck-Hitachi L-7100 pump, an L-7400 UV detector, and a GM tube for radioactivity detection (VWR International) were mounted. The isocratic mobile phase of HPLC comprised 5% acetonitrile and 95% phosphoric acid solution (0.01 M) at a rate of 1 mL/min. Constant monitoring of the HPLC liquid flow was accomplished with a combination of a UV absorbance detector (λ = 210 nm) and a radioactive detector (BETA-flow, Beckman, Fullerton, CA).

Molar radioactivity: This test is based upon UV/Vis spectrophotometric measurement with an absorption-calibrating system for a ligand in molar activity at wavelength 263 nm, where it is marked by the derivatives of the radioactivity of the radioligand in GBq and the mass of the carrier in µmol.

Sterility Testing: The sterility test of [^11^C]KIn83 was performed after decay by an approved contractor using a direct inoculation method. The used method is compliant with regulatory standards to confirm the absence of viable microorganisms in the final product.

Documentation, Record Keeping, and Reporting: Thorough documentation was maintained for all QC tests, results, and actions taken in accordance with GMPs and regulatory requirements to ensure traceability and accountability.

### 3.4. Whole-Body PET Measurements in NHP

This research study followed ethical principles and was approved by the Animal Ethics Committee in Stockholm of The Swedish Board of Agriculture (10367-2019). Also, the study complied with directives stated in the document “Guidelines for planning, conducting and documenting experimental research” (Dnr 4820/06–600) of the Karolinska Institutet. The management of the non-human primates (NHPs) that participated in this study was conducted at the Astrid Fagraeus Laboratory (AFL) of Comparative Medicine located in Solna, Sweden. In this study, two cynomolgus monkeys (NHP1: 5.8 kg and NHP2: 4.6 kg) were used. Anesthesia was induced via an intramuscular injection of sevoflurane (approximately 10 mg/kg) at the AFL and maintained by the administration of a mixture of sevoflurane (2.0–8.0%), oxygen, and medical air with endotracheal intubation. The monkeys’ body temperature was controlled with a Bair Hugger model 505 (Arizant Healthcare, MN), and an esophageal thermometer was used to monitor their temperature. Heart rate, blood pressure, respiratory rate, and oxygen saturation were monitored throughout all the experimental procedures and their values are presented. Fluid balance was provided through the constant infusion of normal saline solution. The Mediso MultiScan^TM^ LFER150 PET/CT (Mediso Ltd., Budapest, Hungary) was utilized for full-body scans. [^11^C]KIn83 was injected intravenously before the dynamic whole-body PET scan covering the head to proximal thigh of the NHPs. The images were reconstructed (2 × 100 s, 3 × 200 s, 4 × 400 s, 4 × 800 s, 1 × 1600 s) through a 3D ordered-subset algorithm with point spread function correction. CT data were acquired for attenuation and scatter correction.

Regions of interest (ROIs) were selected as the brain, heart, liver, lungs, kidneys, gallbladder, bone (lumbar vertebrae), urinary bladder, stomach, small intestine, spleen and pancreas using the PET data, aided with the CT scan for anatomical landmarks. The time–activity curve is represented in terms of percentage of the injected dose (%ID) as follows: (decay-corrected radioactivity in Bq/cc multiplied by ROI volume in cc, over the injected dose in Bq) multiplied by 100.

The estimates of the absorbed dose of radiation in humans were calculated using the OLINDA/EXM 1.1 (Organ-Level Internal Dose Assessment Code) software based on the gender-average adult model [21]. The fractional uptake in NHP organs was assumed to be equal to the uptake in human organs.

## 4. Conclusions

This study represents a step forward in the development of [^11^C]Kln83 as a potential clinical PET tool for investigating the role of α7-nAChR in various diseases and conditions. By addressing both GMP compliance and dosimetry, we lay a strong foundation for the safe and successful clinical application of this promising radioligand.

## Figures and Tables

**Table 1:** Volume (cc) of the regions of interest.

Region of Interest	NHP 1	NHP 2
Brain	61	73
Lungs	106	124
Kidneys	22	27
Spleen	8	6
Gall Bladder	3	12
Liver	114	96
Heart	29	32
Urinary Bladder	23	3
Intestine	391	489
Vertebrae	6 (93)	5 (74)
Thyroid	0.5	1
Salivary Glands	4	3

**Table 2:** The numbers of disintegrations in the source organs for organs in each animal and their means for [^11^C]KIn83.

Region of Interest	NHP 1	NHP 2	Average
Brain	0.00566	0.00491	0.00528
Lungs	0.01343	0.01192	0.01267
Kidneys	0.01546	0.03546	0.02546
Spleen	0.00252	0.00147	0.00200
Gall Bladder	0.00282	0.03805	0.02044
Liver	0.06469	0.05895	0.06182
Heart	0.00483	0.00600	0.00542
Urinary Bladder	0.00969	0.00136	0.00553
Intestine	0.05216	0.03639	0.04428
Vertebrae	0.00984	0.00755	0.00870
Thyroid	0.00056	0.00028	0.00042
Salivary Glands	0.00125	0.00064	0.00094

**Table 3:** Estimated radiation does in different organs.

Target Organ	Alpha	Beta	Gamma	Total	ICRP-103 ED
Adrenals	0.00	1.45 × 10^−3^	4.79 × 10^−3^	6.24 × 10^−3^	5.76 × 10^−5^
Brain	0.00	8.45 × 10^−4^	8.24 × 10^−4^	1.67 × 10^−3^	1.67 × 10^−5^
Breasts	0.00	1.10 × 10^−3^	1.20 × 10^−3^	2.30 × 10^−3^	2.76 × 10^−4^
Esophagus	0.00	1.00 × 10^−3^	2.10 × 10^−3^	3.10 × 10^−3^	1.24 × 10^−4^
Eyes	0.00	1.00 × 10^−3^	9.02 × 10^−4^	1.90 × 10^−3^	0.00
Gallbladder Wall	0.00	4.42 × 10^−2^	1.39 × 10^−2^	5.81 × 10^−2^	5.36 × 10^−4^
Left colon	0.00	1.00 × 10^−3^	3.07 × 10^−3^	4.07 × 10^−3^	1.97 × 10^−4^
Small Intestine	0.00	1.68 × 10^−2^	4.77 × 10^−3^	2.16 × 10^−2^	1.99 × 10^−4^
Stomach Wall	0.00	1.01 × 10^−3^	2.41 × 10^−3^	3.43 × 10^−3^	4.11 × 10^−4^
Right colon	0.00	1.01 × 10^−3^	3.27 × 10^−3^	4.28 × 10^−3^	2.08 × 10^−4^
Rectum	0.00	1.00 × 10^−3^	2.07 × 10^−3^	3.07 × 10^−3^	7.06 × 10^−5^
Heart Wall	0.00	2.42 × 10^−3^	2.44 × 10^−3^	4.86 × 10^−3^	4.49 × 10^−5^
Kidneys	0.00	1.89 × 10^−2^	6.35 × 10^−3^	2.53 × 10^−2^	2.33 × 10^−4^
Liver	0.00	8.62 × 10^−3^	5.63 × 10^−3^	1.43 × 10^−2^	5.70 × 10^−4^
Lungs	0.00	2.58 × 10^−3^	1.93 × 10^−3^	4.51 × 10^−3^	5.42 × 10^−4^
Ovaries	0.00	1.10 × 10^−3^	2.44 × 10^−3^	3.54 × 10^−3^	1.41 × 10^−4^
Pancreas	0.00	1.03 × 10^−3^	3.76 × 10^−3^	4.79 × 10^−3^	4.42 × 10^−5^
Prostate	0.00	9.03 × 10^−4^	1.78 × 10^−3^	2.68 × 10^−3^	1.24 × 10^−5^
Salivary Glands	0.00	2.58 × 10^−3^	1.26 × 10^−3^	3.84 × 10^−3^	3.84 × 10^−5^
Red Marrow	0.00	1.25 × 10^−3^	1.77 × 10^−3^	3.03 × 10^−3^	3.63 × 10^−4^
Osteogenic Cells	0.00	1.23 × 10^−3^	1.62 × 10^−3^	2.86 × 10^−3^	2.86 × 10^−5^
Spleen	0.00	3.15 × 10^−3^	2.80 × 10^−3^	5.95 × 10^−3^	5.49 × 10^−5^
Testes	0.00	9.03 × 10^−4^	1.01 × 10^−3^	1.91 × 10^−3^	7.65 × 10^−5^
Thymus	0.00	1.03 × 10^−3^	1.68 × 10^−3^	2.70 × 10^−3^	2.49 × 10^−5^
Thyroid	0.00	4.76 × 10^−3^	1.60 × 10^−3^	6.36 × 10^−3^	2.54 × 10^−4^
Urinary Bladder Wall	0.00	4.37 × 10^−3^	2.18 × 10^−3^	6.55 × 10^−3^	2.62 × 10^−4^
Uterus	0.00	1.10 × 10^−3^	2.71 × 10^−3^	3.80 × 10^−3^	1.76 × 10^−5^
Total Body	0.00	1.65 × 10^−3^	1.51 × 10^−3^	3.16 × 10^−3^	0.00
Effective Dose					4.77 × 10^−3^

**Table 4:** Specification and analysis of [^11^C]KIn83 solution for injection.

Test Procedure	Acceptance Criteria	Method Reference
pH ^a^	4.5–8.5	pH indicator paper
Radioactivity ^a^	To be reported	Dose calibrator
Product identification ^a^	R_t_ Radiopeak—R_t_ UV ≤ 0.30 min	HPLC
Radiochemical purity ^a^	Not less than 95%	HPLC
Molar activity ^a^	To be reported	HPLC
Chemical amount of KIn83 ^a^	The mass does not exceed 20 µg per injected dose ^b^	HPLC
Total amount of UV-absorbing impurities including precursor ^a^	The mass does not exceed 20.0 µg per injected dose ^b^	HPLC
Filter integrity ^a^	Millex GV not less than 3.5 bar	Bubble point test
Sterility ^c^	Sterile according to Ph Eur	Ph Eur
Bacterial endotoxins ^d^	Less than 14.5 IU/mL	Endosafe PTS
Residual solvents ^d^		
Acetone	Not more than 50 mg	GC
Ethanol	Not more than 100000 ppm	GC
Acetonitrile	Not more than 4.1 mg	GC
DMF	Not more than 8.8 mg	HPLC
Radionuclidic identity ^e^	Half-life 18.3 to 22.4 min	Dose calibrator
Radiochemical stability ^e^	Not less than 95% radiochemical purity after 60	HPLC
Appearance ^f^	Clear and colorless solution free from visual particulates	Visual inspection
Settle plate ^d^	0 CFU	Ph Eur

^a^ Performed on every batch of the product. Results are reported pre-release. ^b^ The injected dose is defined as the maximum injectable volume in order to comply with the mass specification. ^c^ Performed on at least every tenth batch or every third month and all verification and validation batches. ^d^ Performed on every batch of the product. Results are reported post-release. ^e^ Performed on process validation batches only. ^f^ Performed on process validation and verification batches.

## Data Availability

All the supporting data are stored in the Karolinska Institutet archive.

## Supplementary Materials

The supporting information can be downloaded at: https://www.mdpi.com/article/10.3390/molecules30061356/s1. Figure S1: Time activity curves of different organs for NHP 1 and NHP 2; Table S1: Batch analyses for [^11^C]KIn83 Solution for injection; Figure S2: The HPLC chromatogram of the [^11^C]KIn83 solution for injection; Figure S3: The GC chromatogram of the [^11^C]KIn83 solution was used for qualitative and quantitative analysis; Figure S4: The HPLC chromatogram of the [^11^C]KIn83 solution for qualitative and quantitative assessment of DMF.
