# Supplementary material for: Good Manufacturing Practice Validation and Radiation Dosimetry for the Clinical Application of a Novel α7-nAChR Radioligand: [11C]KIn83
Source: Molecules. 2025 Mar 18;30(6):1356. doi: 10.3390/molecules30061356 (PMC11945799; doi:10.3390/molecules30061356)
Supplement: Supplementary file 1 [file molecules-30-01356-s001.zip › molecules-3464490-supplementary.pdf]

Figure S1

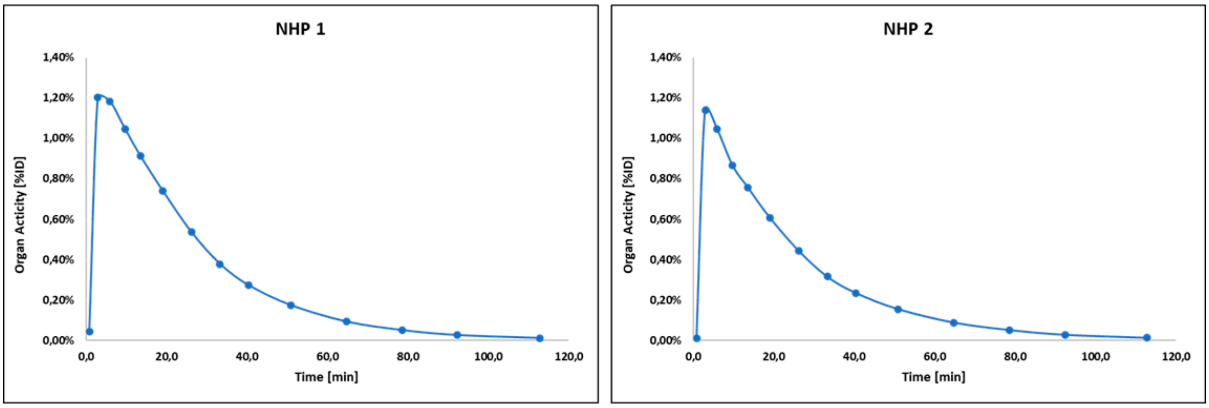

Figure S1a: Time activity curves of brains for NHP 1 and NHP 2

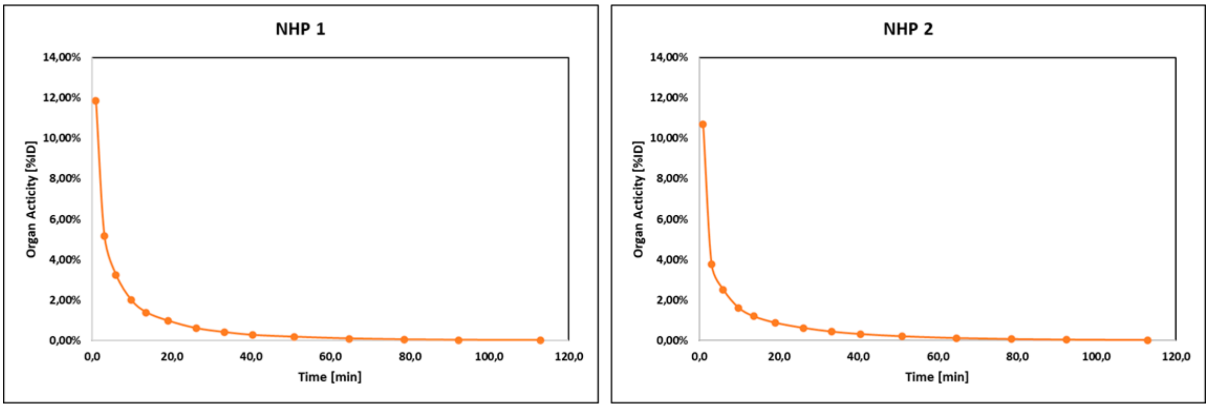

Figure S1b: Time activity curves of lungs for NHP 1 and NHP 2

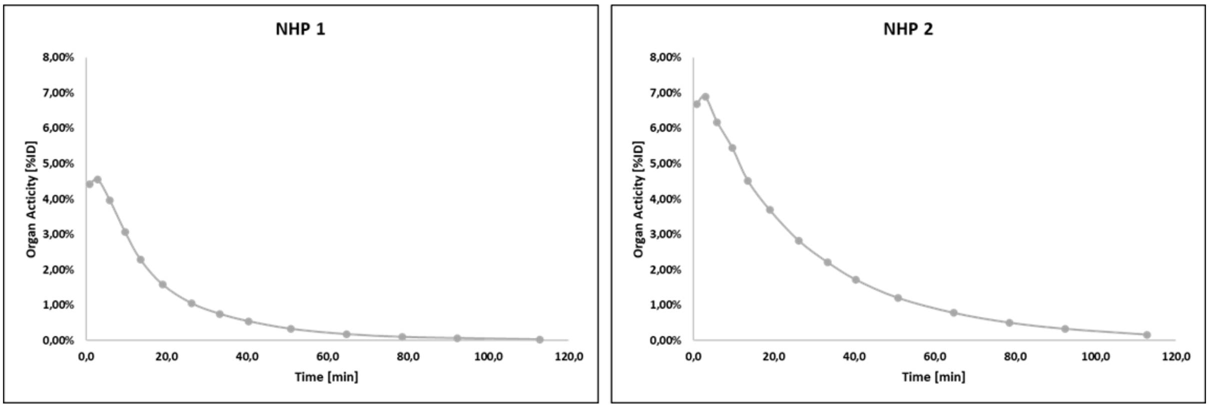

Figure S1c: Time activity curves of kidneys for NHP 1 and NHP 2

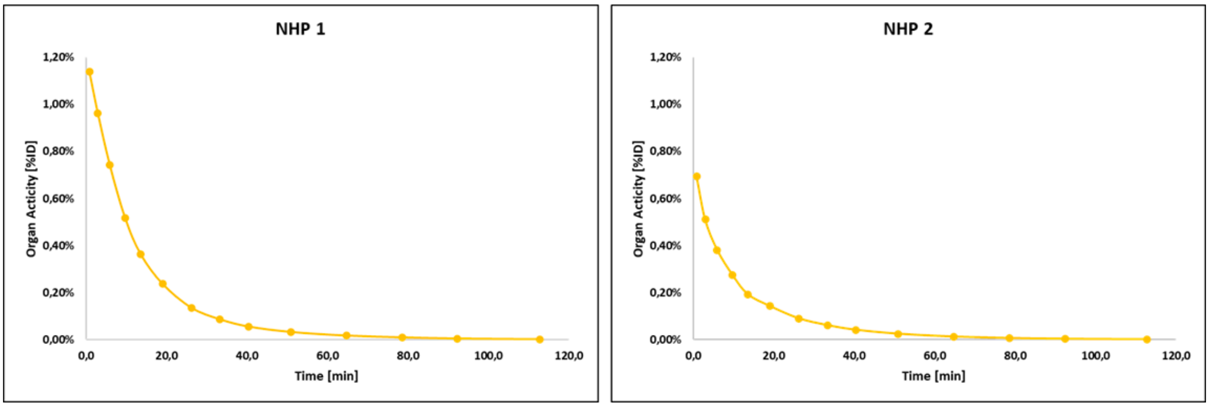

Figure S1d: Time activity curves of Spleens for NHP 1 and NHP 2

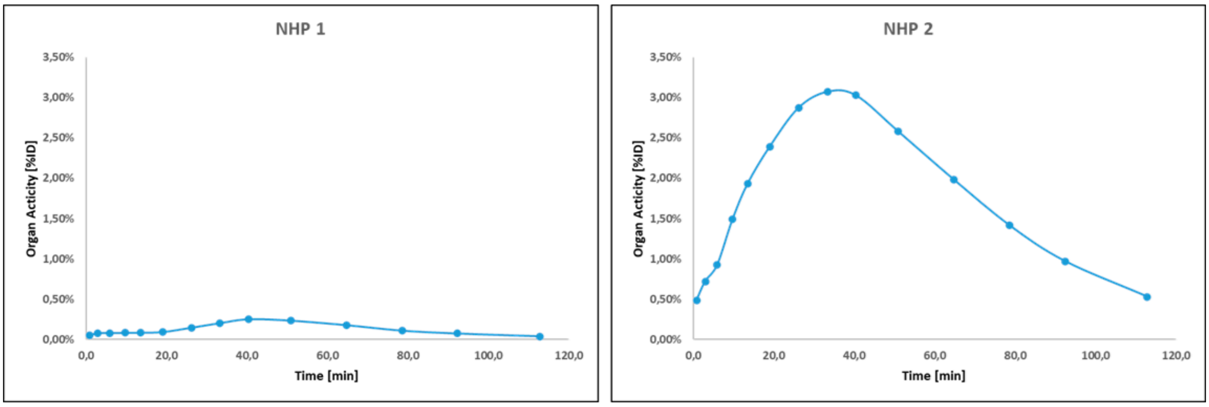

Figure S1e: Time activity curves of gall bladders for NHP 1 and NHP 2

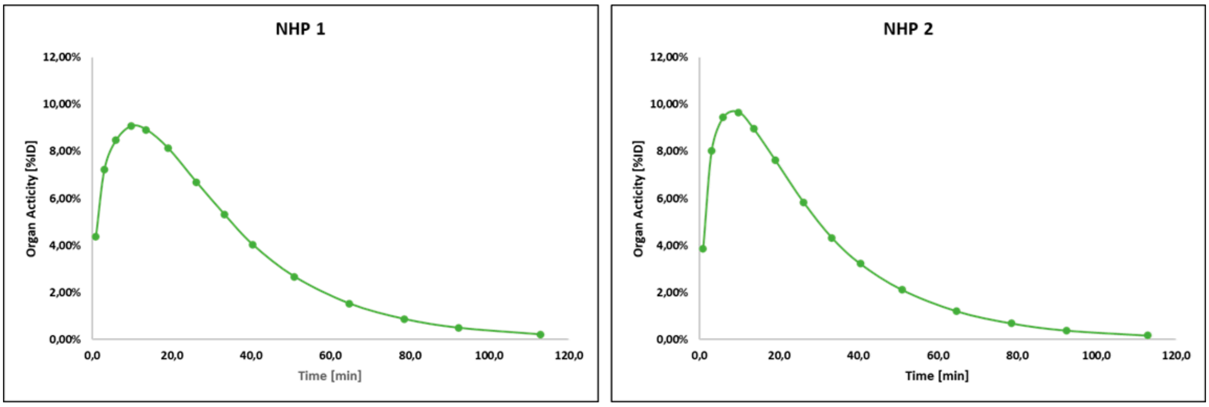

Figure S1f: Time activity curves of livers for NHP 1 and NHP 2

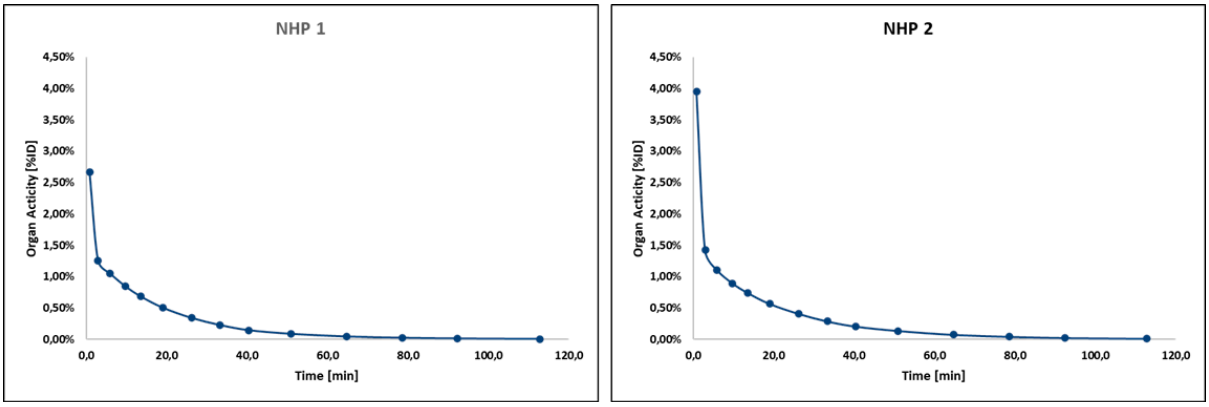

Figure S1g: Time activity curves of hearts for NHP 1 and NHP 2

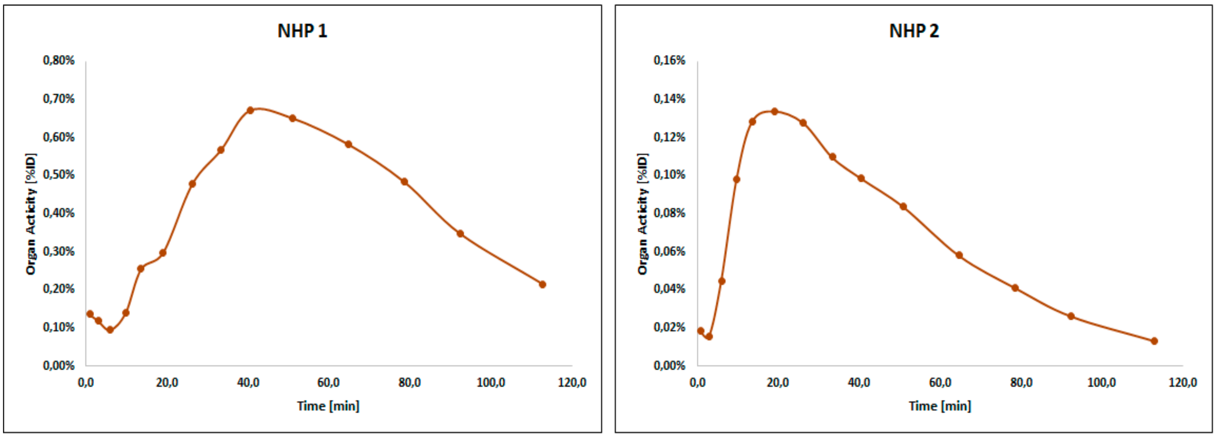

Figure S1h: Time activity curves of urinary bladders for NHP 1 and NHP 2

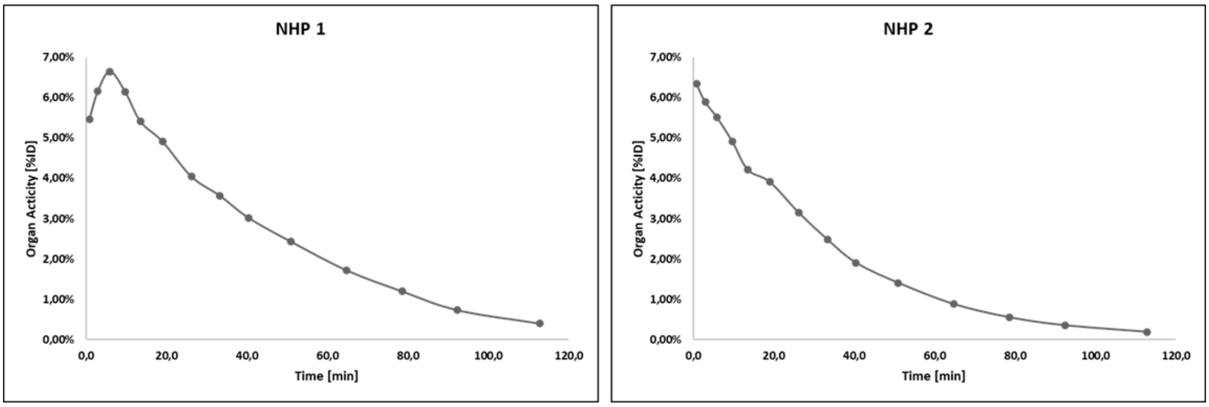

Figure S1i: Time activity curves of intestines for NHP 1 and NHP 2

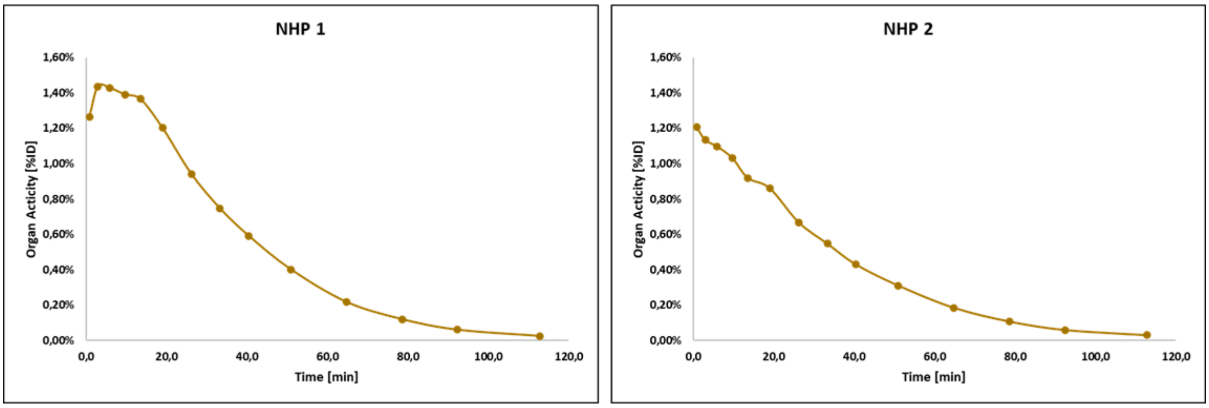

Figure S1j: Time activity curves of vertebraes for NHP 1 and NHP 2

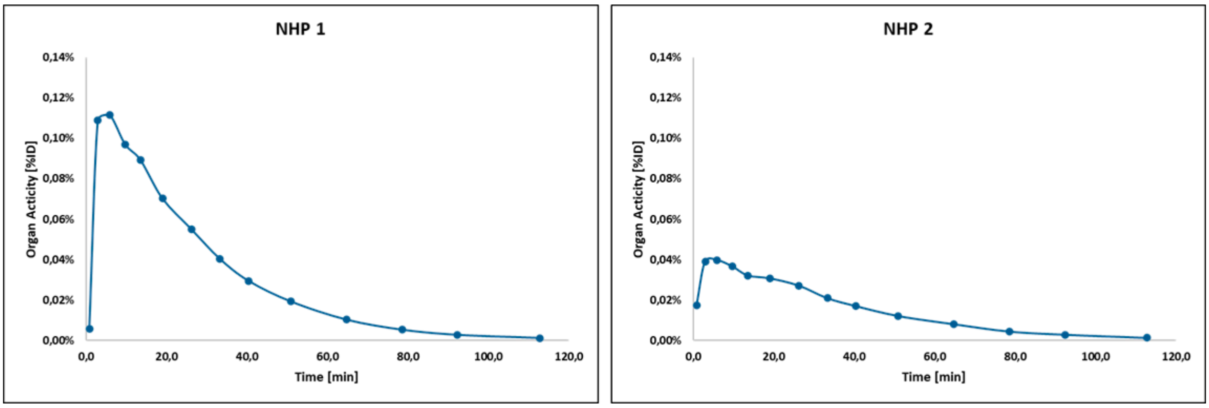

Figure S1k: Time activity curves of throids for NHP 1 and NHP 2

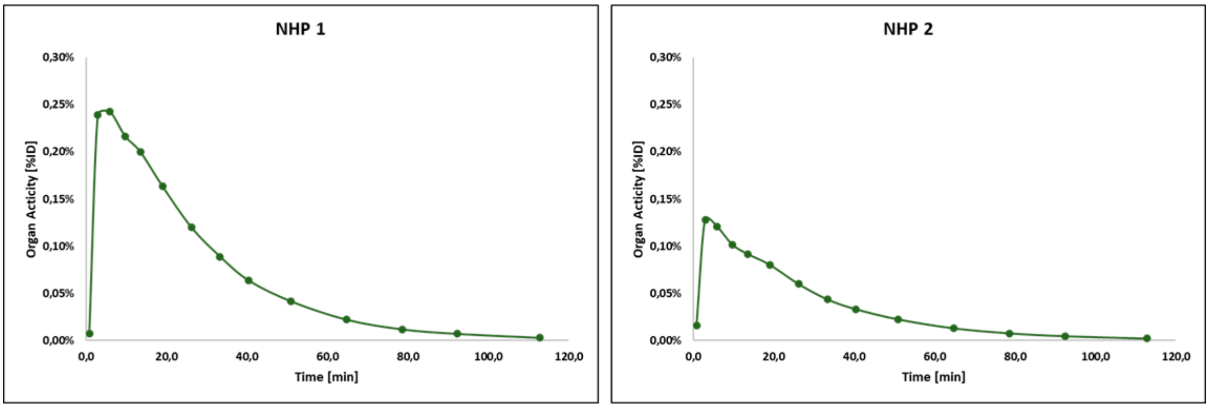

Figure S1l: Time activity curves of salivary glands for NHP 1 and NHP 2

Table S1. Batch analyses for [^{11}C]KIn83 Solution for injection

Purpose		Validation 1	Validation 2	Validation 3	Bioburden
Produced activity		650 MBq	697 MBq	520 MBq	452 MBq
Test	Acceptance criteria	Results	Results	Results	Results
pH	4.5-8.5	5.0	5.0	5.0	5.0
Radionuclidic identity	Half-life 18.3 to 22.4 min	20.4 min	20.6 min	20.4 min	20.4 min
Product identification	R_t Radiopeak-R_t UV less than 0.30 min	0.06 min	0.07 min	0.08 min	0.08 min
Radiochemical purity HPLC	≥ 95%	100%	100%	100%	100%
Chemical amount of KIn83[a]	<20 µg	<10 µg	<10 µg	<10 µg	<10 µg
Total amount of UV-absorbing impurities[a]	<20 µg	<5 µg	<5 µg	<5 µg	<5 µg
Filter integrity	≥ 3.5 bar	3.8 bar	3.8 bar	3.9 bar	N.A.[b]
Sterility	Sterile	Sterile	Sterile	Sterile	Sterile
Bacterial endotoxins	< 14.5 EU/mL	< 3.5 EU/mL	< 3.50 EU/mL	< 3.50 EU/mL	< 3.5 EU/mL
Residual acetonitrile[c]	≤ 4.1 mg	Below LOQ	Below LOD	Below LOQ	Below LOD
Residual acetone[d]	≤ 50.0 mg	Below LOD	10.9 mg	Below LOD	Below LOD
Residual ethanol	≤ 100000 ppm	47108 ppm	49954 ppm	54544 ppm	63940 ppm
DMF content[e]	≤ 8.8 mg	Below LOD	Below LOD	Below LOD	Below LOD
Radiochemical Stability	RCP ≥ 95% after 30 and 60 minutes	100%, 100%	100%, 100%	100%, 100%	N.A.[b]
Visual Inspection	Clear solution, free from visual particulates	Pass	Pass	Pass	Pass
Settle Plates	0 CFU	0 CFU	0 CFU	0 CFU	0 CFU

[a] The limit of detection (LOD) for KIn83 is 0.05 µg/mL. The limit of quantification (LOQ) for KIn83 is 0.16 µg/mL.

[b] Not applicable. Sterile filtration and radiochemical stability test were omitted in the bioburden batch.

[c] The limit of detection (LOD) for acetonitrile is 8 ppm. The limit of quantification (LOQ) for acetonitrile is 25 ppm.

[d] The limit of detection (LOD) for acetone is 111 ppm. The limit of quantification (LOQ) for acetone is 337 ppm.

Figure S2

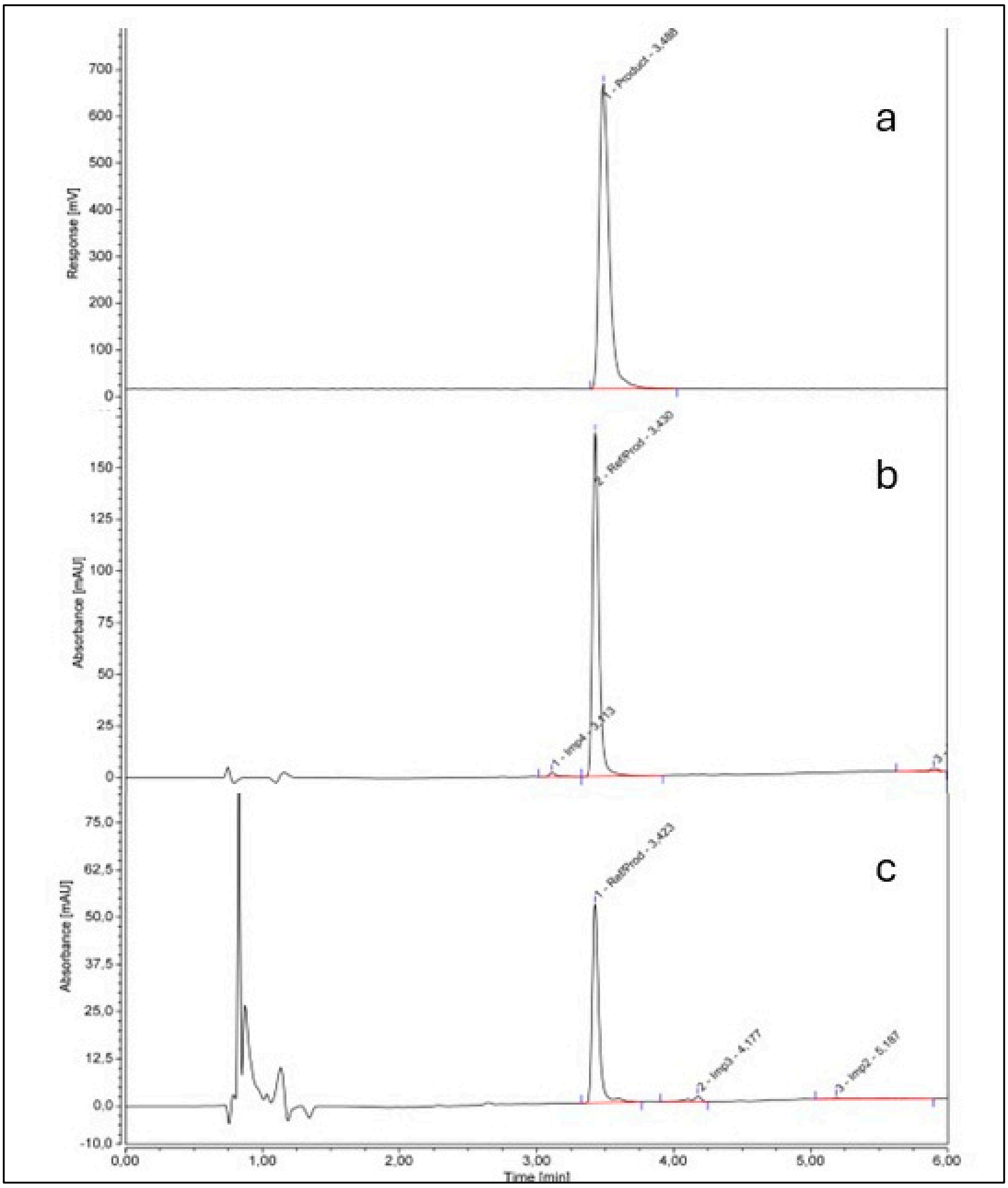

Figure S2. The HPLC chromatogram of the [^{11}C]KIn83 solution for injection presents the following: (a) a radiochemical purity of 100% for [11C]KIn83 at a retention time of 3.488 minutes, measured with a radioactive detector; (b) the reference standard solution of KIn83; and (c) the KIn83 carrier, which elutes at 3.423 minutes, along with impurities that elute at 4.177 and 5.187 minutes in the [^{11}C]KIn83 solution. The total mass of impurities was determined by comparing their UV absorption to that of the reference solution, assuming they share the same UV absorption coefficient.

Figure S3

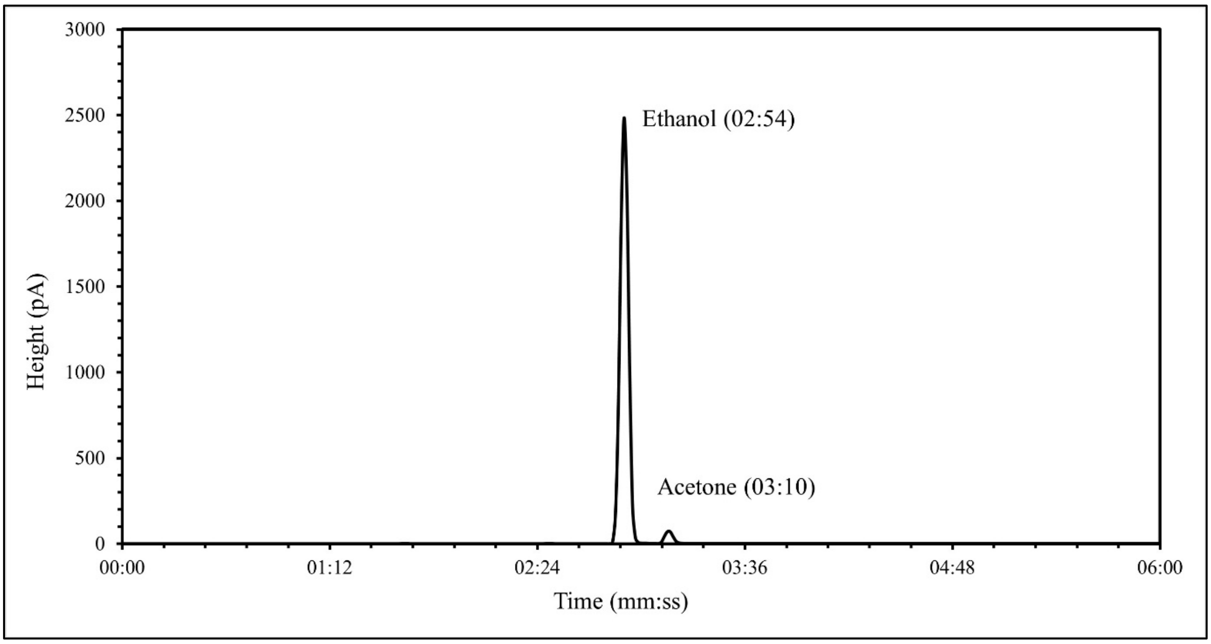

Figure S3. The GC chromatogram of the [^{11}C]KIn83 solution was used for qualitative and quantitative analysis of residual solvents present in the product post-release. Ethanol was detected at a retention time of 02:54 (mm:ss), and acetone was identified at 03:10 (mm:ss). According to the ICH Guideline for Residual Solvents Q3C(R8), amounts of ethanol exceeding the specified limit for class 3 solvents (50 mg) may be acceptable if they are justifiable based on manufacturing capabilities and good manufacturing practices. Ethanol is a critical component of the GMP process for producing the [^{11}C]KIn83 solution for injection and is an integral part of the formulation. The ethanol used in the production of the [^{11}C]KIn83 solution for injection yields a concentration that should not exceed 100,000 ppm; however, the measured ethanol concentration in the [^{11}C]KIn83 solution was below the specified limit (see Table S1). Additionally, based on the measured area counts and a prepared calibration curve, the acetone concentration was determined to be 1,318.7 ppm (reported as total acetone mass in Table S1), which is below the allowable limit for acetone as a class 3 solvent according to the ICH Guideline for Residual Solvents Q3C(R8) ($\leq$ 50 mg per day, corresponding to 5,000 ppm).

Figure S4

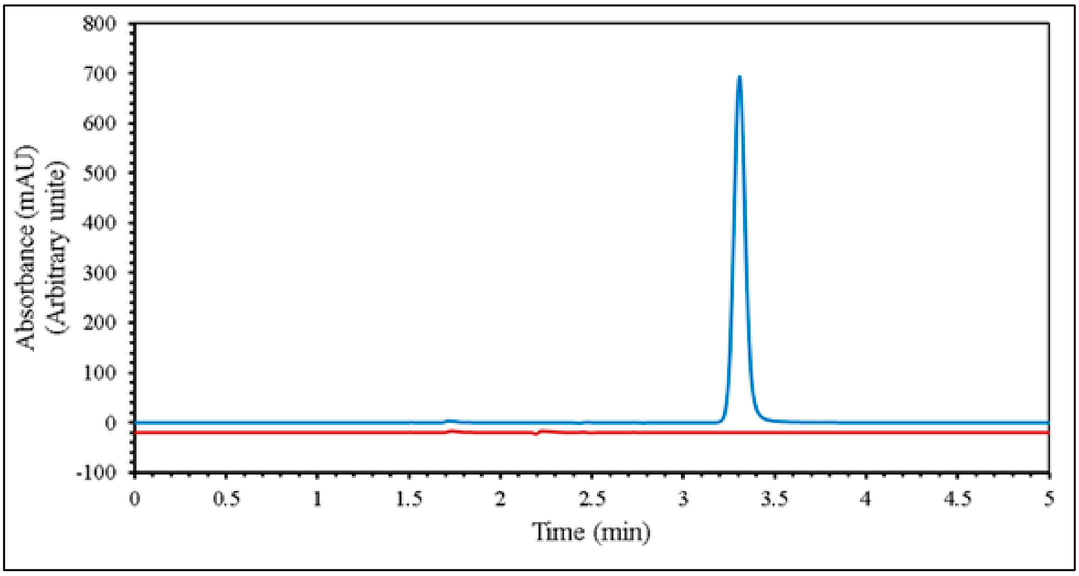

Figure S4. The HPLC chromatogram of the [^{11}C]KIn83 solution was analyzed at a wavelength of 210 nm for qualitative and quantitative assessment of DMF in the product post-release. DMF was not detected at the specified retention time of 3.3 minutes (red line), which corresponds to the retention time of the reference DMF solution compared to the UV signal of the DMF standard solution (blue line). According to the ICH Guideline for Residual Solvents Q3C(R8), the permissible amount of DMF as a class 2 solvent is ≤ 8.8 mg per day, equating to a concentration limit of 880 ppm. The limit of quantification for DMF is 12 ppm.
